# Wilms tumor in a left pelvic kidney: A case report

**DOI:** 10.1016/j.ijscr.2019.11.041

**Published:** 2019-11-27

**Authors:** Adewale Olaotan Oyinloye, Samuel Wabada, Auwal Mohammed Abubakar, Lateef O. Oyebanji, C.U. Rikin

**Affiliations:** a Division of Paediatric Surgery, Department of Surgery, Federal Medical Center, Yola, Adamawa State, Nigeria; b Paediatric Surgery Unit, Department of Surgery, University of Maiduguri Teaching Hospital, Maiduguri, Borno State, Nigeria

**Keywords:** Wilms tumor, Pelvic kidney, Abdominal mass, Chemotherapy, Case report

## Abstract

**Introduction:**

Wilms tumor(nephroblastoma) is the most common renal tumor in childhood.The most frequent anomalies and syndromes associated with this tumor involve the genitourinary tract. The occurrence of Wilms tumor with renal ectopia is rare.When present,Wilms tumor is usually found in association with other forms of renal ectopia like horseshoe kidneys or crossed fused renal ectopia. However, reports of the occurrence of Wilms tumor in unilateral pelvic renal ectopia is uncommon. We report a case of Wilms tumor in a pelvic left kidney.

**Presentation of case:**

A 10 year old girl presented to the out-patient department with a one year history of lower abdominal mass, pain and hematuria. Examination revealed a mass in the suprapubic region. Radiological investigations showed a tumor in a left pelvic kidney. She was managed with preoperative chemotherapy, surgery and adjuvant chemotherapy. Histopathologic examination of the nephrectomy specimen confirmed the diagnosis of nephroblastoma.

**Discussion:**

The occurrence of Wilms tumor in association with renal ectopia is rare. There are few reports of tumors arising in unilateral pelvic ectopic kidneys. A multi-modal treatment approach involving the use of chemotherapy, surgery and radiotherapy (for high-risk tumors) in the management of Wilms tumor gives good outcome.

**Conclusion:**

Tumors arising from a pelvic kidney should be considered as part of the differential diagnosis of lower abdominal masses in children.

## Highlights

• Nephroblastoma is the most common pediatric renal tumor.
• Genitourinary anomalies and syndromes frequently co-exist.
• The existence of Wilms tumor in association with unilateral pelvic renal ectopia is very rare.
• Nephroblastoma in a left pelvic kidney presenting as lower abdominal mass in a 10 year old is presented.

## 1 Introduction

Wilms tumor(nephroblastoma) is the most common solid renal tumor in childhood, with survival rates over 85 % in high-income countries [1]. The majority of anomalies and syndromes associated with Wilms tumor involve the genitourinary tract, including renal ectopia, ureteral duplication, renal hypoplasia, cryptorchidism, hypospadias, and disorder of sexual development [2]. Though generally rare, Wilms tumor is more commonly reported in association with other forms of renal ectopia like horseshoe kidneys or crossed fused renal ectopia than with pelvic renal ectopia [3,4].

We present a rare case of nephroblastoma associated with a left ectopic kidney in a 10-year-old child successfully treated with preoperative chemotherapy followed by surgery and adjuvant chemotherapy. This work has been reported in accordance with the SCARE criteria [5].

## 2 Case report

A 10 year old girl presented at the out-patient department with a one year history of progressively increasing lower abdominal mass with associated episodes of frank haematuria. She complained of dull aching pain which developed a few weeks prior to presentation. There were no other gastrointestinal or genitourinary symptoms. The child had experienced weight loss in the preceding few months before presentation.No history of facial or leg swelling, jaundice, cough, fever or bone pains.

On examination, she was not pale, afebrile, and was well hydrated. Her weight was 22 kg. Pulse was 110beats/minute, respiratory rate 22cycles/minute and blood pressure 110/80 mmHg.Abdominal examination revealed a globular shaped, non-tender suprapubic mass extending slightly to the left iliac fossa measuring about 16 cm × 12 cm. It was firm, could get above and below it, and was slightly mobile. The liver and spleen were not palpably enlarged and the right kidney was not ballotable. Digital rectal examination was unremarkable. A presumptive diagnosis of suspected nephroblastoma in a pelvic kidney to rule out an ovarian teratoma or endodermal sinus tumor was made. Abdominopelvic ultrasonography revealed an empty left renal fossa with a mass in the pelvic lying left kidney. The liver was normal. Serum alpha Fetoprotein(AFP, less than 0.5IU/ml)and liver transaminases were normal but total protein(51 g/l) and albumin(26 g/l) were low.The hematocrit was 30 % and serum electrolytes, urea and creatinine were within normal limits. Urinalysis confirmed hematuria (++). Chest radiograph ruled out pulmonary metastasis.

**Fig. 1:**
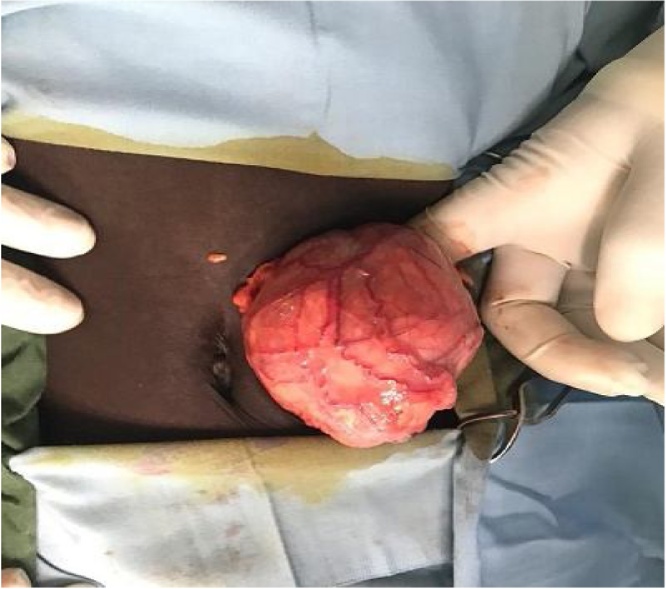
Left pelvic kidney tumor.

CT IVU showed empty left renal bed with an enlarged, malrotated left kidney in the pelvis, superior to the bladder dome. It demonstrated a huge, 9.5 × 10 cm hypodense mass in the mid-portion of the pelvic kidney. There was prompt excretion of contrast from the upper and lower pole calyces. No para-aortic lymph node enlargement was seen. A definitive diagnosis of nephroblastoma in a left pelvic renal ectopia was made. She was commenced on neo-adjuvant chemotherapy(vincristine,actinomycin D) for 6weeks and subsequently scheduled for open (transperitoneal) nephrectomy. Intraoperative findings include a tumor involving the left pelvic kidney situated above the dome of the bladder (Fig. 1). The blood supply arose from the distal abdominal aorta and the left internal iliac artery. There were no para-aortic or hilar lymphadenopathy and no liver or peritoneal deposits.

**Fig. 2:**
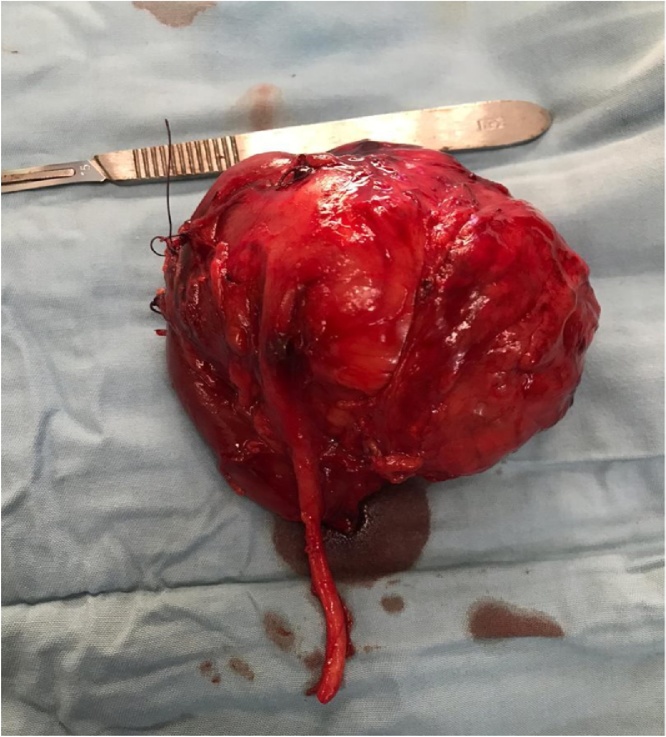
Nephrectomy specimen.

Histopathologic examination showed a 10 × 8.5 × 7 cm nephrectomy specimen weighing 150 g with an intact capsule and a 10 cm long ureter (Fig. 2). Cut surface revealed grey-white to yellowish nodular tumor mass rimmed by renal tissue in some areas. The tumor measured 6.5 cm in its widest diameter. On microscopic section, it was composed of epithelial components in form of abortive tubules and glomeruloid structures. The cells have round to oval hyperchromatic nuclei and abundant cytoplasm. The stroma is abundant and areas of necrosis are present. She did well postoperatively and was commenced on adjuvant chemotherapy prior to discharge. She is currently three months post nephrectomy and being followed up closely.

## 3 Discussion

Pelvic kidney is a rare form of renal ectopia that occurs with an estimated incidence of 1 in 2200 to 1 in 3000 autopsies. There is a slight preponderance on the left side [6]. Renal ectopia is due to the failure of ascent of the kidney from the pelvis during intra-uterine development. The proposed hypotheses to explain this condition include abnormalities of the ureteric bud and metanephric blastema, genetic variants, teratogenic effect, and anomalous vasculature physically blocking ascent [7].

Wilms tumor is the most common renal tumor in childhood, with survival rates over 85 % in high-income regions of the world [1]. However, management of this condition remains challenging in developing countries [8]. The majority of anomalies and syndromes associated with Wilms tumor involve the genitourinary tract, including renal ectopia, ureteral duplication, renal hypoplasia, cryptorchidism, hypospadias, and disorder of sexual development [2]. Though rare, Wilms tumor is more commonly associated with other forms of renal ectopia like horseshoe kidneys or crossed fused renal ectopia than with pelvic renal ectopia [3,4].

There have been few reports written on malignancies in a pelvic kidney [[9], [10], [11], [12], [13]]. Even more infrequent are reports of Wilms tumor in association with pelvic kidney as the majority of these reports were from adult patients and the tumors were histologically confirmed as renal cell carcinoma. Sarin and colleagues reported a case of recurrent monophasic Wilms tumor in the left pelvic kidney in a 1 year old boy [14]. To the best of our knowledge, this will be the second report in English literature of nephroblastoma in a unilateral pelvic kidney. The treatment of Wilms tumor is multimodal, involving surgery and chemotherapy, with the addition of radiotherapy for children with high-risk tumors [15].

The administration of neo-adjuvant chemotherapy reduced the tumor size by more than 30 % in the index patient. She was successfully managed with a combination of surgery and chemotherapy.

In conclusion, tumors arising from a pelvic kidney should be considered as part of the differential diagnosis of lower abdominal masses in children.

## Funding

No source of funding for this study.

## Ethical approval

The study is exempt from ethical approval in our institution.

## Consent

Full informed consent was obtained from the parents of the patient.

## Author contribution

Adewale O Oyinloye: Conceptualization and design of paper.

Adewale O Oyinloye, Samuel Wabada and Auwal M Abubakar; Manuscript drafting and review.

Lateef O Oyebanji, Chrsitopher Rikin: Case summary, study design and image acquisition.

All authors read and approved the final manuscript.

All authors fully participated in the care of the patient.

## Registration of research studies

Not applicable.

## Guarantor

Adewale O Oyinloye stands as the guarantor for this study.

## Provenance and peer review

Not commissioned, externally peer-reviewed.

## Declaration of Competing Interest

All authors have no conflict of interest to declare.
